# Preliminary investigation on the effect of insect-based chitosan on preservation of coated fresh cherry tomatoes

**DOI:** 10.1038/s41598-023-33587-0

**Published:** 2023-04-29

**Authors:** Elena Tafi, Micaela Triunfo, Anna Guarnieri, Dolores Ianniciello, Rosanna Salvia, Carmen Scieuzo, Annamaria Ranieri, Antonella Castagna, Samuel Lepuri, Thomas Hahn, Susanne Zibek, Angela De Bonis, Patrizia Falabella

**Affiliations:** 1 Department of Sciences, University of Basilicata, Potenza, Italy; 2 Spinoff XFLIES s.r.l, University of Basilicata, Potenza, Italy; 3 Department of Agriculture, Food and Environment, University of Pisa, Pisa, Italy; 4 Interdepartmental Research Center Nutrafood “Nutraceuticals and Food for Health”, University of Pisa, Pisa, Italy; 5 Fraunhofer Institute for Interfacial Engineering and Biotechnology IGB, Stuttgart, Germany

**Keywords:** Biomaterials, Entomology

## Abstract

Chitosan was produced from *Hermetia illucens* pupal exuviae by heterogeneous and homogeneous deacetylation. Tomato fruits (*Solanum lycopersicum*), that are one of the most grown and consumed food throughout the world, were coated with 0.5 and 1% chitosan, applied by dipping or spraying, and stored at room temperature or 4 °C, for a storage period of 30 days. Statistical analysis give different results depending on the analysed parameters: heterogeneous chitosan, indeed, had a better effect than the homogenous one in maintaining more stable physico-chemical parameters, while the homogenous chitosan improved the total phenols, flavonoids and antioxidant activity. Chitosan coatings applied by spraying were more effective in all the analyses. Chitosan derived from *H. illucens* always performed similarly to the commercial chitosan. However, a general better performance of insect-derived chitosan on the concentration of phenolics and flavonoids, and the antioxidant activity was observed as compared to the commercial one. Chitosan coating has already been successfully used for preservation of fresh fruits, as alternative to synthetic polymers, but this is the first investigation of chitosan produced from an insect for this application. These preliminary results are encouraging regarding the validation of the insect *H. illucens* as a source of chitosan.

## Introduction

Tomato (*Solanum lycopersicum*) is one of the most widely grown and consumed fruits throughout the world, due to its convenience of use and content of health-promoting compounds^1^. Tomatoes are perishable climacteric fruits, which continue to undergo respiration and biochemical ripening mechanisms in the post-harvest life, leading to senescence and deterioration^2^. Packaging is thus a key element in preserving the postharvest quality of tomatoes. Many different methods have been used to preserve quality of fresh food, especially fruits and vegetables, and enhance their shelf life, including low temperature storage, modified atmosphere packaging, and chemical treatments^3^. In recent years research has focused on the development of preservation and packaging technologies with less environmental impact than conventional packaging based on the use of synthetic polymers. Edible coatings, directly applied on the product surface, based on natural biodegradable polymers (e.g., polysaccharides, proteins, lipids, of vegetable or animal origin) are among the most successful alternatives^4^. Chitosan, the main deacetylated derivative of chitin (a structural component of the arthropods’ exoskeleton), is one of the most promising natural polymers for use as an edible coating, due to its antimicrobial^5^, antioxidant and film forming ability. Chitosan-based coatings can act as barriers, able to delay maturation and senescence, reduce dehydration, and retard microbial and fungal spoilage, and they have already been used successfully on a variety of fresh fruit and vegetables^6,7^. Traditionally, chitosan is produced at an industrial level by alkaline hydrolysis of chitin extracted from the waste exoskeletons of crustaceans processed for human consumption^8^. In the last decade, increasing efforts have been made to find alternative sources of chitin, driven by an exponential growth in the market for this polymer. Insects are among the alternatives that have received the most attention, due to the increasing availability of chitin-rich biomass generated as waste from large-scale insect farms aimed at feed production that have been developed worldwide^9^. Among farmed insects, *Hermetia illucens* L. (the black soldier fly) is the most bred species in Europe, due to its ability of bioconverting organic waste into protein-, fat-, bioactive compound-rich biomass, usable in feed, energetic, cosmetic and pharmaceutical fields^10–19^. The main waste biomass of *H. illucens* breeding is the exuviae generated by the fly during the transition from pupa to the adult stage. These pupal exuviae contain up to 25% chitin and they are therefore a viable starting material for the production of chitosan. Through chemical methods, chitin can be extracted from the pupal exuviae of *H. illucens* by demineralization with acids and deproteinization with sodium hydroxide. Afterwards, the deacetylation treatment using highly concentrated alkali provides the production of chitosan^20,21^.

Chitosan, suitably functionalized, can be used to create film for food preservation by overlaying the surface of the food product with the biopolymer solution. This coating, with its excellent characteristics, can be applied through different methods^22,23^. The easiest and most economical way to prepare chitosan coating is by its solubilization in a slightly acidic aqueous solution. Plasticizer agents (e.g., glycerol, Tween 80) are then generally added to the solution to enhance its viscosity and adhesion properties^24^. These chitosan solutions can be directly applied to food products in their liquid state. In most cases food products are coated with chitosan solution by dipping followed by drying under flowing air^25,26^, or by spraying the solution on their surface^22,27^. The aim of this preliminary work was to provide, for the first time, an investigation regarding the ability of an insect-derived chitosan to act as a protective barrier for maintaining the post-harvest quality of fresh cherry tomatoes. The objective was to evaluate the validity of chitosan obtained from a new sustainable source to be used as an alternative to the commercially available polymer for application as edible coating.

## Results

### Chitin and chitosan characterization

Chitin and chitosan structure was examined by FTIR spectroscopy to identify the characteristic bands. Spectra of unbleached and bleached chitin extracted from pupal exuviae presented all the bands at the specific wavelengths and showed a structural similarity with the commercial polymer (Supplementary Fig. 1a(A–B_1_)). The α-form was assigned to all chitin samples, detecting the split of the amide I band into two peaks at 1650 and 1620 cm^−1^^28^. In the FTIR spectra of all chitosan samples, the characteristic bands at 1650 cm^−1^ (amide I) and 1590 cm^−1^ (amide II) were recognized, confirming the chitosan formation after chitin deacetylation^29^; the structural similarity between chitosan from pupal exuviae and the commercial sample was also observed (Supplementary Fig. 1b(A–C_1_)). According to Kumirska et al*.*^28^, homogeneous unbleached chitosan appeared less deacetylated than the heterogeneous one, since the band at 1590 cm^−1^ (NH_2_ bending) had a lower intensity than the one at 1655 cm^−1^ (amide I) (Supplementary Fig. 1b(A–C_1_)).

Data on chitosan characterization are reported in Table 1. Bleached chitosan samples had a deacetylation degree (DD) similar to the commercial polymer, while the heterogeneous unbleached one was slightly less deacetylated. The lower deacetylation of the homogeneous unbleached sample was confirmed. Viscosity-average molecular weight (M_v_) of insect chitosan, especially the homogeneous bleached sample, was much lower than those of the commercial sample (Table 1).

**Table 1:** Deacetylation degree (DD) and viscosity-average molecular weight (M_v_) of heterogeneous and homogeneous chitosan samples obtained from both unbleached and bleached chitin, and the commercial sample.

Chitosan sample	DD (%)	M_v_ (kDa)
Heterogeneous unbleached	82 ± 1.2	76 ± 1.4
Heterogeneous bleached	88 ± 0.8	68 ± 0.5
Homogeneous unbleached	62 ± 2.8	84 ± 0.2
Homogeneous bleached	70 ± 1.4	46 ± 2.0
Commercial	93 ± 0.8	370 ± 1.1

Data are expressed as mean ± standard deviation.

Supplementary Fig. 2 shows the results of the chitosan filmogenic ability assessment. All chitosan samples were able to form a film, uniform in surface and thickness, with no holes or damaged areas, strong enough to be removed from the Petri dishes and handled without breaking.

The viscosity values of the coating solutions (Supplementary Table 1) followed a similar trend to the M_v_ of the respective chitosan samples. Given the same chitosan concentration, coatings made with insect chitosan were less viscous than those prepared with the commercial polymer. Furthermore, the chitin bleaching treatment led to the formation of a less viscous chitosan solution. The solvent alone coating had the lowest viscosity.

### Effect of chitosan-based coatings

#### Weight loss

**Figure 1:**
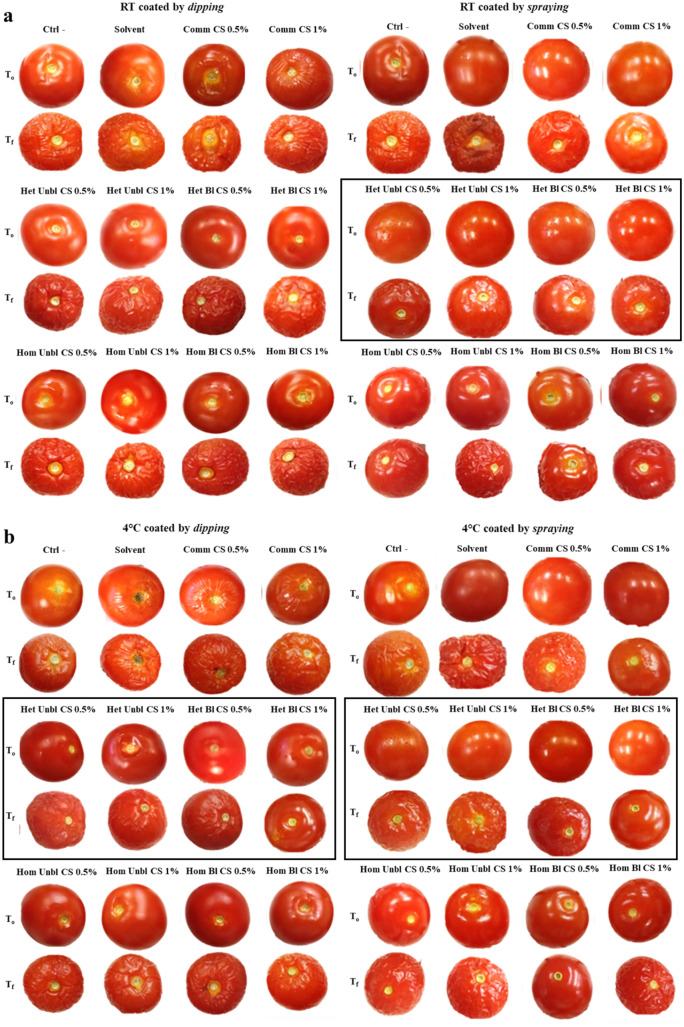
Pictures of tomatoes coated by dipping and spraying at the beginning (T_0_) and after 30 days (T_f_) of storage period at (**a**) room temperature (RT) and (**b**) 4 °C. Treatments that gave the best results are in black frames. Treatments: untreated fruits (Ctrl-), solvent, coating with 0.5 and 1% of commercial chitosan (Comm CS), heterogeneous unbleached (Het Unbl CS), heterogeneous bleached (Het Bl CS), homogeneous unbleached (Hom Unbl CS), homogeneous bleached (Hom Bl CS) chitosan from *H. illucens* pupal exuviae.

In dipping-coated tomatoes stored at both temperatures (Fig. 1a), no treatment was effective in reducing weight loss in comparison with the untreated fruits (Table 2). At room temperature (RT), tomatoes coated with heterogeneous chitosan had a weight loss similar to the negative control, but significantly lower than the solvent control. Homogeneous chitosan gave a weight loss higher or similar to both controls. At 4 °C, the lowest weight loss was observed in the negative control, and in tomatoes coated with the 1% heterogeneous unbleached chitosan, 0.5% heterogeneous bleached and the 1% homogeneous unbleached one (Table 2). All the other chitosan treatments gave results similar to both controls and to each other.

**Table 2:** Results of evaluation of weight loss, total soluble solids (TSS) and pH on whole tomatoes, coated by dipping, after 30 days of storage at room temperature (RT) and 4 °C.

Treatments	Parameters		
	Weight loss (%)	TSS (°Brix)	pH
Before treatment	–	6.2 ± 0.5	4.17 ± 0.04
After storage at RT			
Ctrl -	30 ± 1.8^cd^	6.9 ± 0.1^cde^	4.52 ± 0.07^bc^
Solvent	38 ± 2.7^ab^	8.4 ± 0.6^ab^	4.53 ± 0.06^bc^
Comm CS 0.5%	35 ± 2.6^bc^	8.0 ± 0.0^abc^	4.56 ± 0.04^ab^
Comm CS 1%	35 ± 3.3^bc^	8.0 ± 0.1^abc^	4.55 ± 0.01^b^
Het Unbl CS 0.5%	32 ± 2.5^c^	7.9 ± 0.1^de^	4.31 ± 0.05^c^
Het Unbl CS 1%	28 ± 4.3^cd^	8.5 ± 0.1^cde^	4.40 ± 0.02^cd^
Het Bl CS 0.5%	28 ± 2.9^cd^	9.0 ± 0.1^bcde^	4.43 ± 0.01^c^
Het Bl CS 1%	26 ± 3.9^d^	7.5 ± 0.7^e^	4.68 ± 0.02^a^
Hom Unbl CS 0.5%	36 ± 4.8^bc^	9.6 ± 0.3^ab^	4.39 ± 0.08^ab^
Hom Unbl CS 1%	37 ± 1.9^b^	8.2 ± 0.3^bcde^	4.40 ± 0.07^cd^
Hom Bl CS 0.5%	35 ± 3.3^bc^	8.5 ± 0.1^bcde^	4.25 ± 0.02^d^
Hom Bl CS 1%	42 ± 4.4^a^	10.2 ± 0.2^a^	4.33 ± 0.02^bc^
After storage at 4 °C			
Ctrl -	18 ± 1.3^c^	7.0 ± 0.0^ab^	4.36 ± 0.08^b^
Solvent	25 ± 3.5^b^	6.7 ± 0.1^ab^	4.42 ± 0.03^ab^
Comm 0.5%	24 ± 1.3^b^	7.1 ± 0.1^ab^	4.71 ± 0.07^a^
Comm 1%	26 ± 2.3^ab^	7.1 ± 0.2^ab^	4.49 ± 0.02^a^
Het Unbl CS 0.5%	25 ± 2.5^b^	8.1 ± 0.4^ab^	4.28 ± 0.03^bc^
Het Unbl CS 1%	20 ± 3.1^c^	7.9 ± 0.1^ab^	4.30 ± 0.04^bc^
Het Bl CS 0.5%	20 ± 3.1^c^	7.9 ± 0.1^ab^	4.25 ± 0.04^c^
Het Bl CS 1%	29 ± 1.5^a^	8.0 ± 0.0^ab^	4.44 ± 0.02^ab^
Hom Unbl CS 0.5%	30 ± 2.6^ab^	8.2 ± 0.1^ab^	4.20 ± 0.02^bc^
Hom Unbl CS 1%	22 ± 3.1^bc^	7.9 ± 0.2^ab^	4.14 ± 0.02^c^
Hom Bl CS 0.5%	31 ± 2.4^ab^	7.9 ± 0.3^ab^	4.05 ± 0.03^d^
Hom Bl CS 1%	36 ± 3.4^a^	8.7 ± 0.1^a^	4.05 ± 0.03^d^

Treatments: untreated fruits (Ctrl -), solvent only, coating with heterogeneous unbleached (Het Unbl CS), heterogeneous bleached (Het Bl CS), homogeneous unbleached (Hom Unbl CS), homogeneous bleached (Hom Bl CS) and commercial (Comm CS) chitosan. Means followed by different letters in the column are significantly different by Mann–Whitney U test (p < 0.05). Each trial contained three triplicates of whole tomatoes per each treatment. Asterisks indicate significant differences (*p < 0.05; **p < 0.01) between the two storage temperatures within the same treatment, according to Mann–Whitney U test.

Also in spraying-coating tomatoes (Fig. 1b), no chitosan treatment was effective in significantly reducing the weight loss compared to the negative control, irrespective of the storage temperature (Table 3). However, all chitosan coatings significantly reduced the fruit weight loss compared to the solvent control, at both storage conditions (except for treatment with 1% homogeneous bleached chitosan at RT and 1% commercial chitosan at 4 °C). At RT, tomatoes coated with all the homogeneous chitosan solutions lost significantly more weight than those treated with heterogeneous chitosan, while at 4 °C no differences were observed (Table 3).

**Table 3:** Results of evaluation of weight loss, total soluble solids (TSS) and pH on whole tomatoes, coated by spraying, after 30 days of storage at room temperature (RT) and 4 °C.

Treatments	Parameters		
	Weight loss (%)	TSS (°Brix)	pH
Before treatment	–	6.5 ± 0.6	4.05 ± 0.01
After storage at RT			
Ctrl -	29.9 ± 1.8^bc^	6.9 ± 0.1^e^	4.52 ± 0.1^ab^
Solvent	59.3 ± 4.7^a^	9.6 ± 0.6^c^	4.48 ± 0.04^a^
Comm CS 0.5%	28.2 ± 4.6^bc^	8.7 ± 0.5^e^	4.24 ± 0.02^bc^
Comm CS 1%	28.9 ± 2.4^bc^	9 ± 0^de^	4.24 ± 0.1^bc^
Het Unbl CS 0.5%	25.6 ± 4.1^c^	8.4 ± 0.3^e^	4.04 ± 0.1^d^
Het Unbl CS 1%	26.2 ± 3.1^c^	9.9 ± 0.1^cd^	4.04 ± 0.05^d^
Het Bl CS 0.5%	26.0 ± 4.2^c^	8.8 ± 0.3^e^	4.28 ± 0.1^b^
Het Bl CS 1%	23.8 ± 3.6^c^	8.8 ± 0^e^	4.15 ± 0.1^bc^
Hom Unbl CS 0.5%	30.6 ± 2.8^b^	10.6 ± 0.2^bc^	4.25 ± 0.03^b^
Hom Unb CS 1%	33.1 ± 1.9^b^	10.8 ± 0.3^abc^	4.29 ± 0.03^ab^
Hom Bl CS 0.5%	30.9 ± 3.3^b^	11.0 ± 0.3^ab^	4.21 ± 0.06^bc^
Hom Bl CS 1%	40.1 ± 2.3^a^	11.6 ± 0.3^a^	4.08 ± 0.05^c^
After storage at 4 °C			
Ctrl -	18.6 ± 2.5^d^**	7 ± 0^d^	4.36 ± 0.08^ab^
Solvent	52.3 ± 4.6^a^	10.9 ± 0.1^a^*	4.69 ± 0.02^a^
Comm CS 0.5%	22.0 ± 4.9^cd^	9.2 ± 0^bcd^	4.12 ± 0.01^b^
Comm CS 1%	30.1 ± 4.4^a^	9.5 ± 0.1^bc^	4.20 ± 0.01^ab^
Het Unbl CS 0.5%	28.1 ± 3.7^bc^	9.7 ± 0.1^bc^*	3.91 ± 0.01^d^
Het Unbl CS 1%	24.9 ± 2.8^bc^	9.1 ± 0.1^bcd^*	3.87 ± 0.02^d^*
Het Bl CS 0.5%	22.2 ± 2.4^c^	9.8 ± 0.3^bc^*	4.05 ± 0.04^bc^*
Het Bl CS 1%	19.6 ± 3.3^cd^	9.0 ± 0^ cd^	4.10 ± 0.02^b^
Hom Unbl CS 0.5%	23.9 ± 2.5^c^**	9.8 ± 0.1^bc^*	4.04 ± 0.05^bc^*
Hom Unbl CS 1%	23.8 ± 3.1^c^**	9.9 ± 0.2^b^*	4.06 ± 0.04^bc^*
Hom Bl CS 0.5%	23.5 ± 2.2^c^**	9.7 ± 0.2^bc^**	4.09 ± 0.01^c^*
Hom Bl CS 1%	21.4 ± 3.2^c^**	9.2 ± 0^bcd^**	4.08 ± 0.06^bc^

Treatments: untreated fruits (Ctrl-), solvent only, coating with heterogeneous unbleached (Het Unbl CS), heterogeneous bleached (Het Bl CS), homogeneous unbleached (Hom Unbl CS), homogeneous bleached (Hom Bl CS) and commercial (Comm CS) chitosan. Means followed by different letters in the column are significantly different by Mann–Whitney U test (p < 0.05). Each trial contained three triplicates of whole tomatoes per each treatment. Asterisks indicate significant differences (*p < 0.05; **p < 0.01) between the two storage temperatures within the same treatment, according to Mann–Whitney U test.

**Figure 2:**
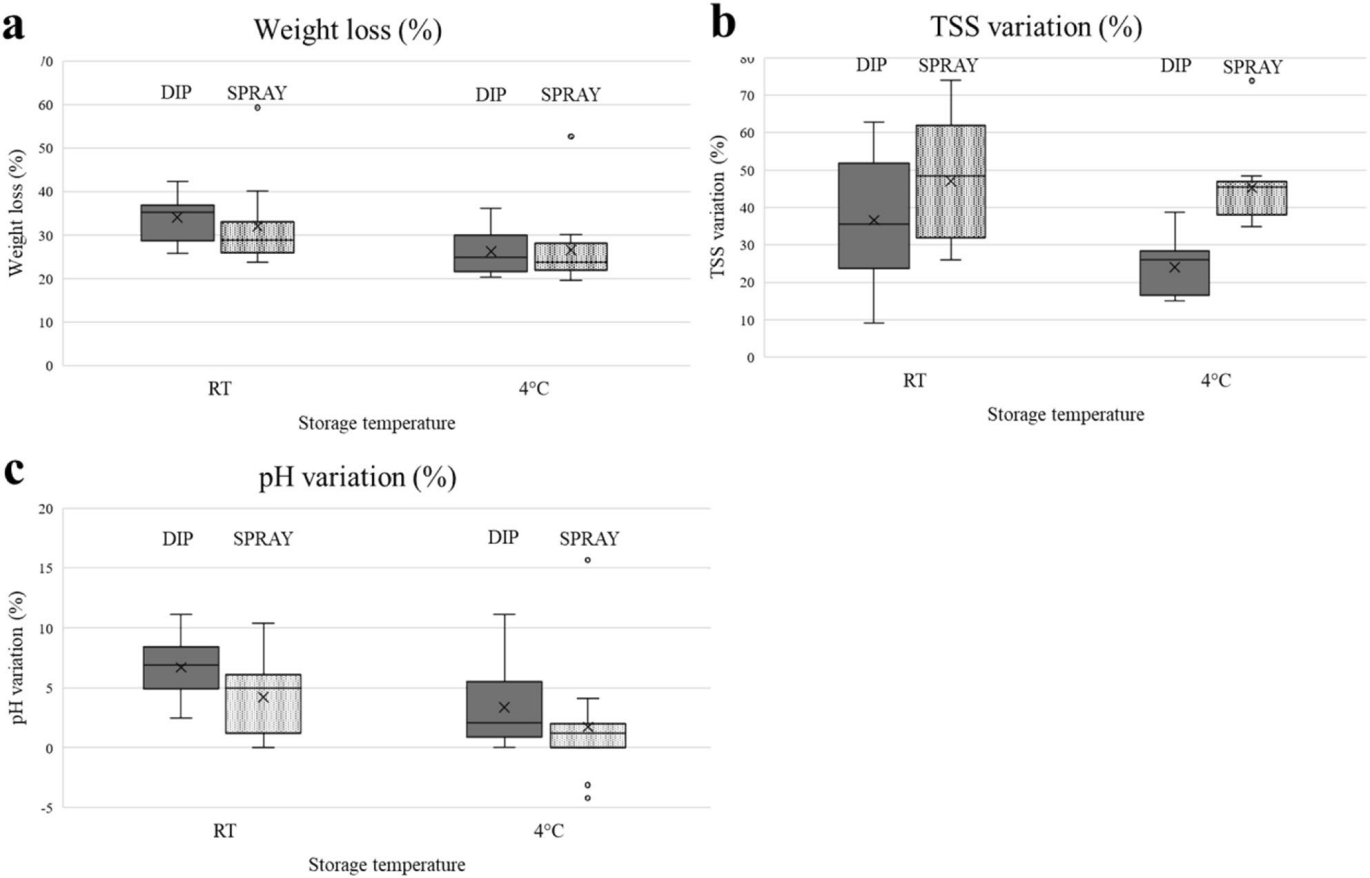
Box plots representing the variation in weight loss (**a**), TSS content (**b**) and pH (**c**) of tomatoes depending on the coating application method (dip or spray).

Comparing the effect of the coating application method, weight loss was reduced overall in sprayed tomatoes compared to the dipped fruits, at both storage temperatures (Fig. 2a). Details of statistically significant comparisons are provided in Supplementary Table 2.

#### Total soluble solids variation

The total soluble solids (TSS) of tomatoes increased in all treatments for both storage temperature conditions, regardless of the coating application method.

In dipped tomatoes stored at RT, no chitosan treatment significantly reduced the TSS increase compared to the negative control (Table 2). Heterogeneous chitosan gave a lower TSS increase than the solvent alone, having a better effect than the homogeneous samples. No differences among treatments were observed in dipped tomatoes stored at 4 °C.

Within sprayed tomatoes, the lowest TSS variation occurred in the negative control, at both storage temperatures (Table 3). At RT, heterogeneous chitosan gave a TSS increase higher than the negative control, but lower than the solvent alone, being significantly more effective in reducing the TSS variation compared to the homogeneous chitosan. At 4 °C, all chitosan coatings had a similar effect, maintaining TSS more stable than the solvent alone.

Comparing the coating application method, TSS variation was greater in sprayed tomatoes than in the dipped ones, especially at cold storage (Fig. 2b). Detailed results of the statistical analysis are reported in Supplementary Table 3.

#### pH variation

pH of dipping-coated tomatoes stored at RT increased during storage (Table 2). Coating with 0.5% homogeneous unbleached chitosan was the most effective in reducing the pH variation. All the other treatments (except for the 1% heterogeneous bleached chitosan) had a similar effect to the controls. Within fruits stored at 4 °C, pH increased in both controls, and in tomatoes coated with commercial chitosan and 1% heterogeneous bleached chitosan (Table 2). The other treatments with both heterogeneous and homogeneous chitosan maintained a pH stable.

Within spraying-coated tomatoes, heterogeneous unbleached chitosan had generally the best effect, maintaining the pH stable and even decreasing it at 4 °C storage (Table 3). Both homogeneous and commercial chitosan had a worse effect compared to the heterogeneous polymer. The greatest pH rise was observed in both control treatments at both storage temperatures.

The pH had a greater variation in dipping-coated tomatoes than in sprayed ones, regardless of the storage temperature (Fig. 2c). Results of statistical comparisons are provided in detail in Supplementary Table 4.

#### Concentration of total phenolics

Because of the very slight differences induced by chitosan concentration on the above reported parameters, total phenolics and flavonoids and the antioxidant activity were quantified only in fruits coated with 1% chitosan. Dipping-coated tomatoes stored at RT presented the lowest concentration of total phenolic compounds when treated with the solvent solution and the commercial chitosan, as compared to the negative control, that had the highest phenolic content (Table 4). Heterogeneous unbleached chitosan allowed a partial recovery of phenolic concentration, that was fully achieved in fruits coated with homogeneous chitosan, with a better performance by the bleached polymer (Table 4). When fruits were stored at 4 °C, solvent control and heterogeneous bleached chitosan had the lowest phenolic concentration, while homogeneous bleached chitosan resulted to be the best treatment. All the other coatings behaved similarly and induced similar accumulation of phenolics to the negative control (Table 4).

**Table 4:** Results of evaluation of total phenols (TP), total flavonoids (TF) and antioxidant activity (AA) on whole tomatoes, coated by dipping, after 30 days of storage at room temperature (RT) and 4 °C.

Treatments	Parameters		
	TP (mg GAE g^−1^ f.w.)	TF (mg CE g^−1^ f.w.)	AA (μmol TE g^−1^ f.w.)
Before treatment	0.339 ± 0.019	0.043 ± 0.004	0.558 ± 0.056
After storage at RT			
Negative control	0.716 ± 0.108^a^	0.162 ± 0.015^a^	1.499 ± 0.125^a^
Solvent	0.332 ± 0.041^c^	0.060 ± 0.009^c^	0.589 ± 0.125^d^
Comm CS 1	0.299 ± 0.051^c^	0.063 ± 0.001^c^	0.578 ± 0.057^d^
Het Unbl CS 1	0.517 ± 0.053^b^	0.068 ± 0.014^c^	0.667 ± 0.142^cd^
Het Bl CS 1	0.435 ± 0.028^bc^	0.101 ± 0.007^bc^	1.021 ± 0.250^bc^
Hom Unbl CS 1	0.608 ± 0.065^ab^	0.140 ± 0.012^ab^	1.436 ± 0.224^ab^
Hom Bl CS 1	0.696 ± 0.067^a^	0.133 ± 0.039^ab^	1.463 ± 0.008^a^
After storage at 4 °C			
Negative control	0.458 ± 0.043^ab^*	0.081 ± 0.005^a^**	1.191 ± 0.032^ab^*
Solvent	0.287 ± 0.111^b^	0.045 ± 0.016^a^	0.862 ± 0.379^ab^
Comm 1	0.370 ± 0.104^ab^	0.060 ± 0.031^a^	0.802 ± 0.304^ab^
Het Unbl CS 1	0.364 ± 0.051^ab^*	0.070 ± 0.024^a^	0.897 ± 0.256^ab^
Het Bl CS 1	0.309 ± 0.011^b^**	0.041 ± 0.018^a^**	0.548 ± 0.157^b^*
Hom Unbl CS 1	0.484 ± 0.063^ab^	0.081 ± 0.003^a^**	1.215 ± 0.167^a^
Hom Bl CS 1	0.559 ± 0.095^a^	0.058 ± 0.005^a^*	1.361 ± 0.183^a^

Treatments: untreated fruits (negative control), solvent only, coating with heterogeneous unbleached (Het Unbl CS), heterogeneous bleached (Het Bl CS), homogeneous unbleached (Hom Unbl CS), homogeneous bleached (Hom Bl CS) and commercial (Comm CS) chitosan. GAE, gallic acid equivalents. CE, catechin equivalents. TE, Trolox equivalents. Means followed by different letters in the column are significantly different by Mann–Whitney U test (p < 0.05). Each trial contained three triplicates of whole tomatoes per each treatment. Asterisks indicate significant differences (*p < 0.05; **p < 0.01) between the two storage temperatures within the same treatment, according to Mann–Whitney U test.

As for the dipping application, spraying-coated fruits stored at RT showed the highest phenolic concentration in the negative control, and the lowest concentration in the commercial chitosan and in solvent control (Table 5). All the insect-chitosan coatings gave results similar to the negative control, and the homogeneous ones, irrespective of the bleaching step, and the heterogeneous bleached behaved better than the commercial chitosan. In fruits stored at 4 °C the differences among the treatments were less pronounced, with solvent control and homogeneous samples showing the lowest and the highest phenolic concentration, respectively, and all the other treatments having similar and intermediated contents (Table 5).

**Table 5:** Results of evaluation of total phenols (TP), total flavonoids (TF) and antioxidant activity (AA) on whole tomatoes, coated by spraying, after 30 days of storage at room temperature (RT) and 4 °C.

Treatments	Parameters		
	TP (mg GAE g^−1^ f.w.)	TF (mg CE g^−1^ f.w.)	AA (μmol TE g^−1^ f.w.)
Before treatment	0.339 ± 0.019	0.043 ± 0.004	0.558 ± 0.056
After storage at RT			
Negative control	0.716 ± 0.108^a^	0.162 ± 0.015^a^	1.499 ± 0.125^ab^
Solvent	0.442 ± 0.073^bc^	0.074 ± 0.005^b^	0.823 ± 0.106^c^
Comm CS 1	0.333 ± 0.117^c^	0.078 ± 0.028^b^	0.668 ± 0.119^c^
Het Unbl CS 1	0.539 ± 0.057^abc^	0.068 ± 0.006^b^	0.679 ± 0.066^c^
Het Bl CS 1	0.542 ± 0.052^ab^	0.062 ± 0.004^b^	0.594 ± 0.066^c^
Hom Unbl CS 1	0.632 ± 0.003^ab^	0.146 ± 0.001^a^	1.773 ± 0.184^a^
Hom Bl CS 1	0.597 ± 0.050^ab^	0.138 ± 0.015^a^	1.384 ± 0.127^b^
After storage at 4 °C			
Negative control	0.458 ± 0.043^ab^*	0.081 ± 0.005^a^**	1.191 ± 0.032^a^*
Solvent	0.246 ± 0.043^b^*	0.039 ± 0.018^a^*	0.534 ± 0.194^b^
Comm 1	0.295 ± 0.012^ab^	0.057 ± 0.009^a^	0.651 ± 0.105^ab^
Het Unbl CS 1	0.373 ± 0.087^ab^*	0.060 ± 0.019^a^	0.708 ± 0.214^ab^
Het Bl CS 1	0.347 ± 0.110^ab^*	0.046 ± 0.025^a^	0.590 ± 0.228^ab^
Hom Unbl CS 1	0.485 ± 0.102^a^	0.077 ± 0.012^a^**	0.858 ± 0.281^ab^**
Hom Bl CS 1	0.473 ± 0.107^a^	0.059 ± 0.001^a^**	1.120 ± 0.316^ab^

Treatments: untreated fruits (negative control), solvent only, coating with heterogeneous unbleached (Het Unbl CS), heterogeneous bleached (Het Bl CS), homogeneous unbleached (Hom Unbl CS), homogeneous bleached (Hom Bl CS) and commercial (Comm CS) chitosan. GAE, gallic acid equivalents. CE, catechin equivalents. TE, Trolox equivalents. Means followed by different letters in the column are significantly different by Mann–Whitney U test (p < 0.05). Each trial contained three triplicates of whole tomatoes per each treatment. Asterisks indicate significant differences (*p < 0.05; **p < 0.01) between the two storage temperatures within the same treatment, according to Mann–Whitney U test.

A significant reduction due to storage at 4 °C was observed in the negative control, in heterogeneous chitosan, irrespective of bleaching and coating application mode (Tables 4 and 5), and in solvent control, this latter limited to the spraying-coated samples (Table 4).

**Figure 3:**
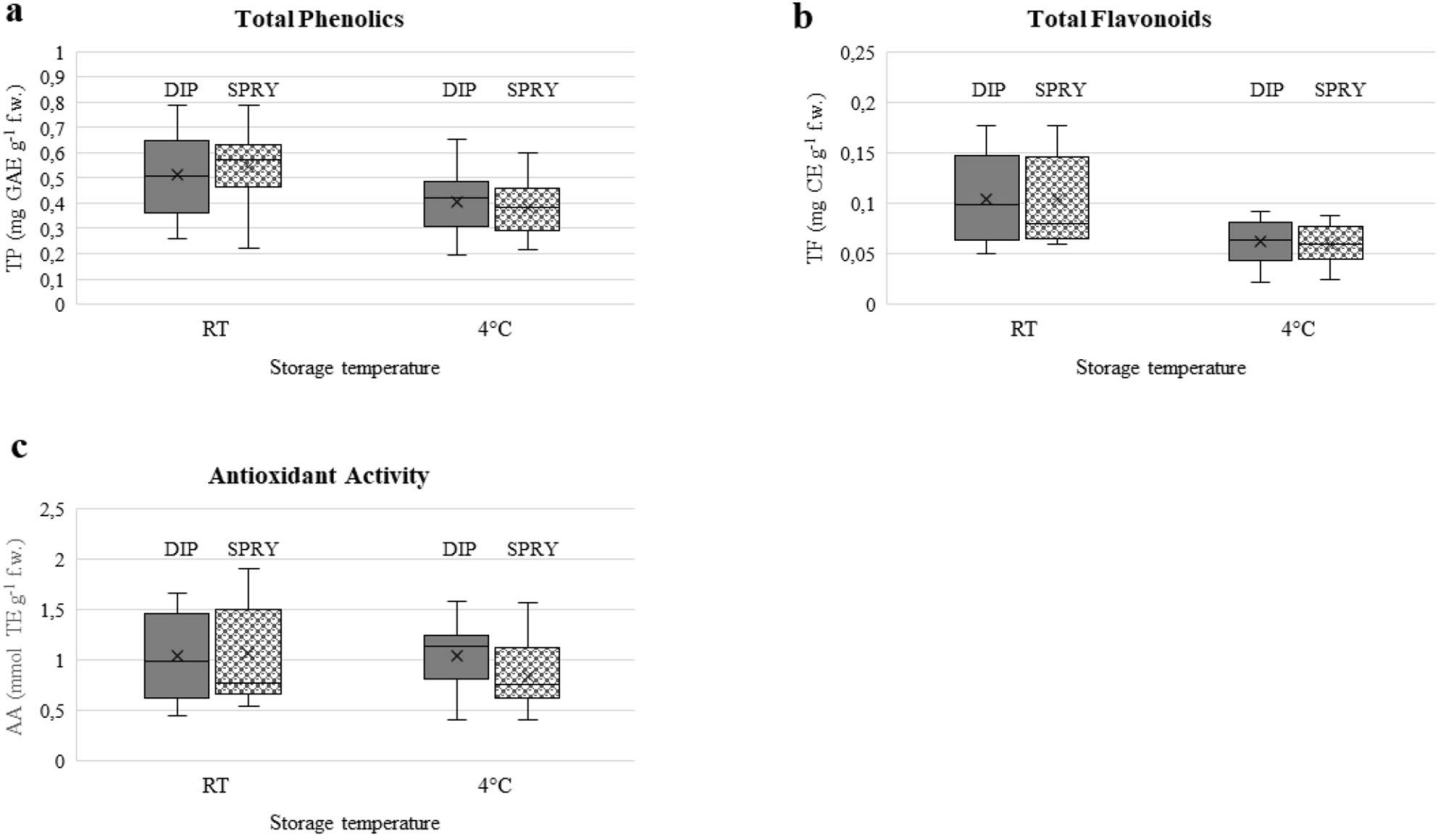
Box plots representing the variation in the concentration of total phenolics (**a**) and total flavonoids (**b**), and in antioxidant activity (**c**) of tomatoes depending on the coating application method (dipping or spray).

Overall, the coating application mode (dipping vs spraying) did not influence the concentration of total phenolics at both storage temperatures (Fig. 3a). However, comparing the two application methods for each treatment at the same storage temperature, some barely significant differences are detected (Supplementary Table 5). In detail the concentration of total phenolics was affected by the coating application mode in commercial chitosan at 4 °C; solvent, heterogeneous bleached and homogeneous bleached chitosan at RT.

#### Concentration of total flavonoids

Total flavonoids of dipping-coated tomatoes at RT were less concentrated in solvent control, commercial chitosan and heterogeneous unbleached chitosan, as compared to the negative control, that displayed the highest concentration (Table 4). Differently from heterogeneous chitosan, homogeneous chitosan, both bleached and unbleached, had flavonoid concentration similar to the negative control. Fruits stored at 4 °C showed no significant variation of flavonoid concentration among the different treatments.

Concerning the spraying application at RT, the highest concentration of total flavonoids was detected in negative control and in both bleached and unbleached homogeneous chitosan. All the other treatments displayed the lowest concentration, without any significant differences among them (Table 5). When fruits were stored at 4 °C, flavonoid concentration was unaffected by the coating treatment.

The influence of storage temperature was evident in negative control, that underwent a decrease in flavonoid concentration at 4 °C as compared to RT. Storage at 4 °C decreased flavonoid concentration also in dipping-coating heterogeneous bleached and homogeneous chitosan (Table 4), as well as in fruits sprayed with homogeneous chitosan, irrespective of its bleaching, compared to fruits subjected to the same treatments but stored at RT (Table 5).

As observed for total phenolics, the application method did not affect the concentration of total flavonoids overall (Fig. 3b). Only in tomatoes treated with solvent and heterogeneous bleached chitosan, both at RT, a significant difference was detected (Supplementary Table 6).

#### Variation in antioxidant activity

At RT, the lowest antioxidant activity in dipping-coated tomatoes was observed in solvent control and in fruits coated with commercial chitosan, while negative control and homogeneous bleached chitosan samples had the highest antioxidant activity. Heterogeneous bleached chitosan behaved better than its unbleached counterpart, though both had lower activity than the negative control (Table 4). Fruits stored at 4 °C displayed the lowest antioxidant activity when coated with heterogeneous bleached chitosan, while the best antioxidant activity was recorded in homogeneous chitosan, irrespective of its bleaching. All the other treatments had similar intermediate values (Table 5).

Spaying-coated fruits stored at RT presented the highest antioxidant activity in homogeneous unbleached chitosan, while solvent control, commercial chitosan and both bleached and unbleached heterogeneous chitosan showed the lowest activity (Table 4). When fruits were stored at 4 °C, solvent control still displayed the lowest activity as compared to the negative control that, instead, was the best performing sample (Table 5).

Storage at 4 °C significantly decreased the antioxidant activity of negative control, of dipping-coated heterogeneous bleached chitosan and of spraying-coated homogeneous unbleached chitosan (Tables 4 and 5).

The antioxidant activity was the parameter most influenced by the coating application method (Fig. 3c). In detail, the coating mode significantly affected the antioxidant activity in all treatments, except for commercial and heterogeneous unbleached chitosan, at RT (Supplementary Table 7). At 4 °C a significant effect of the application method was observed only for treatment with homogeneous bleached chitosan (Supplementary Table 7).

## Discussion

### Chitosan filmogenic ability

One of the most widely used, simple and economical methods for preparing chitosan-based coatings and films is the solution casting method, involving chitosan dissolution in an acidic aqueous solution followed by evaporation. During drying, intermolecular interactions and chain entanglements, including electrostatic and hydrogen bonds, are formed, induced by the increase in the concentration of chitosan. These bonding play an essential role in the structure of polysaccharides and lead to the formation of the chitosan film^30,31^. The ability of the chitosan produced from *H. illucens* to form films validated the use of these samples for the desired application, demonstrating their capacity to form coatings. However, the chitosan-coating properties can be affected by the characteristics of the chitosan itself. The major differences between chitosan produced from *H. illucens* and the commercial polymer were found in viscosity and molecular weight, two of the main factors influencing the properties of the coating solution. Certain effects observed on fruits subjected to the different treatments are therefore discussed in the following paragraphs in relation to characteristics of the chitosan coating used.

### Effect of chitosan-based coatings

#### Weight loss

Chitosan coating has been effective in many cases in reducing the weight loss of fresh tomatoes, whether stored at room or cold temperature^1,7,25,32–34^, as coating confers a physical barrier to moisture loss, retarding dehydration and fruits shrivelling^35,36^. In the present work, the solvent alone treatment caused a greater weight loss than the negative control, while almost all chitosan coatings gave the same weight loss as the negative control, or a lower loss than the solvent alone. Thus, a non-optimal formulation of the solvent solution could be speculated. If this were true, chitosan would have mitigated the detrimental effect of the solvent, improving its performance in reducing tomato weight loss. The composition of the solvent used (i.e., 1% acetic acid, 0.2% Tween-80 and 2% glycerol) is in accordance with the literature^25,26,37,38^, but a modification of the components or their concentrations may be necessary. For instance, it has been shown that the type of plasticizer can affect the mechanical and permeation properties of the chitosan coating. A different plasticizer could therefore be tried instead of glycerol (e.g., sorbitol, propylene glycol)^39^. Additional factors that can have adversely influenced the effect of the coating are the chitosan molecular weight and the viscosity of the resulting solution chitosan, which can affect the adhesion, thickness, and permeability of the coating. In general, the higher the viscosity, the more easily the chitosan solution can adhere to the surface of the treated product and form a thick coating, more effective in extending its shelf life^40,41^. Low molecular weight and viscosity can hinder the formation of a proper barrier on the surface of the treated fruit, thus affecting the moisture-retaining effect of the coating.

#### Total soluble solids variation

The efficacy of crustacean chitosan coatings in reducing the variation of total soluble solids (TSS), has been confirmed for several different fruits, including longan fruit^2^, blueberry^42^, mango^43,44^, papaya^27^, blackberry^45^, lemon^46^ and pomegranate^47^. Results of TSS variation obtained in this work are similar to those obtained by Sucharita et al.^25^ with tomatoes stored at cold temperature for 30 days. They observed a general increase in TSS during storage. Only coating with 0.25% chitosan solution significantly reduced the TSS increase, while 0.5% chitosan solution was not effective^25^. We did not observe significant differences between the two chitosan concentrations, but it might be useful to test other higher and lower concentrations than 0.5 and 1%. In contrast, Barreto et al.^33^ reported a decrease in TSS in tomatoes stored both at room temperature for 12 days and at cold temperature for 24 days. In either condition, coating with chitosan significantly reduced the TSS decrease^33^. The chitosan coating can modify the internal atmosphere of the fruit, with a reduction in the oxygen level and/or an increase in the carbon dioxide level; thus, the respiration rate and metabolic activity are reduced, both the accumulation of sugars and the starch degradation are delayed, and fermentation processes could be started^7,36,43^.

Variation of the TSS content depends on the physiological stage of the tomatoes. According to the literature, the increase in TSS observed both in the present work and by Sucharita et al.^25^ could indicate either tomatoes in the ripening stage or in advanced storage phase. Whereas the decrease in TSS could indicate the physiological phase just after ripening and before the reactivation of ethylene production and respiration^44,48^. Since the exact time of harvest of the tomatoes used in the present work is unknown, it is reasonable to assume that at the time of purchase, being visually ripe, the fruits were already in the phase of reactivation of the biochemical ripening mechanisms, which then led to the constant increase in TSS throughout the storage period.

#### pH variation

During the fruit postharvest storage, acidity usually decreases as a result of the acid metabolism that converts acid and starch into sugars^42^. Thus, an increase in pH of fruits usually occurs with storage. The reduction in the pH increase mediated by chitosan coatings is a sign of a deceleration of the acidity decline due to a slowdown in the acid metabolism of the fruit^42^. The effect of chitosan coating in the present work was more pronounced at cold temperature than at room temperature. It is known that increasing the storage temperature can modify the acid metabolism, by affecting activity of enzymes involved in the glycolysis and the tricarboxylic acid cycle^49^. Generally, with higher temperature the fruit respiration is stimulated and the citrate production during ripening is decreased, thus lowering the fruit acidity. But the final effect on the fruit acidity varies depending on the fruit species and stage of ripeness^49^. An increase in the pH of other different fruits during storage was reported, with a positive effect of chitosan coating in reducing the alkalisation of the fruit^42,50^, except for blackberry investigated by Vilaplana et al.^45^ for which pH increased more in coated fruits than in the control. However, many factors can affect acidity, including fruit variety, cultivation practices, growing area, climate, and transport conditions^49,51^.

#### Concentration of total phenolics

Despite many papers have been published on the effects of chitosan coating on fruit quality, comprising the evaluation of its ability to preserve the phenolic compounds and flavonoids, to the best of our knowledges the research was all carried out using commercial or self-produced crustaceous-derived chitosan. Generally, a positive influence of chitosan coating was reported in different fruits, such as longan fruit^2^, mango^44^, banana^52^, sweet cherry^53^, strawberry^54^ and grape^55^. However, chitosan coating was not effective in preserving the content of phenolic compounds of postharvest strawberries from 1 to 8 days after treatment as compared to untreated controls, unless turmeric or green tea extracts were added to the coating solution^56^. Khatri et al.^57^ found that 2% chitosan coating allowed the preservation of total phenolics for a longer time than control tomatoes and attributed this finding to a slower ripening process in coated fruits. Indeed, when considering a specific time point during storage, phenolics were less concentrated in coated tomatoes than in control ones. A similar conclusion was reported also by Pagno et al*.*^7^ for tomato fruits coated with 1.5% chitosan.

It is interesting noting that, in our experiment, despite the commercial chitosan induced a decrease in phenolic concentration as compared to the negative control, mainly in fruits stored at RT, the insect-derived chitosan was effective in maintaining the phenolic concentration to the same values (at RT) or even higher (at 4 °C) than control ones, depending on the specific kind of chitosan considered. As stated before, an improvement in the formulation of the solvent solution, that markedly affected the phenolic concentration, would result in better performance of these coatings. Nevertheless, the higher efficiency of insect-derived chitosan as compared to the commercial one is undoubted, indicating that insect-derived chitosan stimulates the phenolic biosynthesis. Indeed, increased gene expression of some genes involved in the phenylpropanoid biosynthetic pathway was detected in chitosan-coated avocado fruits^58^.

#### Concentration of total flavonoids

As for phenolic compounds, the current literature reports the effectiveness of chitosan coating in preserving flavonoids during fruit storage. Such an effect, in the pericarp of longan fruits, was evident starting from 3 days after the treatment, though it was observed only for one of the chitosan concentrations used^2^. Riaz et al.^54^ also detected a positive influence of chitosan coating on strawberry flavonoids of up to 6 days storage, particularly marked when chitosan solution was enriched with apple peel polyphenols. Differently from the finding reported above, in our experiment, as already discussed for phenolic compounds, chitosan coating was generally ineffective in maintaining higher concentration as compared to the negative control, because of the negative influence played by the solvent solution. However, also for flavonoids, differences in the effectiveness of commercial and insect-derived chitosan are evident, being homogeneous chitosan able to contrast the solvent-induced decrease of flavonoids observed at room temperature. The activity of chitosan as an elicitor molecule able to activate the plant immunity and to modulate the metabolome of leaf tissues, thanks to its structure very similar to fungal cell wall fragments, is known^59^. We hypothesise that the lower deacetylation degree of the homogeneous chitosan, as compared to the heterogeneous one, may be responsible for its higher activity.

#### Antioxidant activity

The antioxidant activity was often reported to be positively influenced by chitosan coating, such as in banana^52^, strawberry^54^, and grape^55^. In chitosan-coated mango the activity was lower than in control fruit up to 14 days of storage and increased thereafter up to the end of the 33 days storage^44^.

However, in accordance with our findings on insect-derived chitosan, Yang et al*.*^56^ did not observe any increase in the antioxidant activity of chitosan-coated strawberries as compared to untreated controls, and only the addition of green tea extracts improved the efficiency of the coating solution. The antioxidant activity of chitosan-coated tomato fruits was maintained for a longer storage period as compared to the untreated samples Khatri et al.^57^. However, when the activity was tested by the DPPH method, control fruits showed a higher activity that chitosan-coated ones up to 14 days of storage.

The antioxidant activity is an important indicator of the healthy status of the fruit, and it is often well correlated with the concentration of phenolic and flavonoid compounds. Indeed, antioxidant activity displayed a trend similar to those exhibited by these metabolites. Specifically, also for this parameter, in our experiment the superior performance of insect-derived chitosan, namely the homogeneous one, was evident, irrespective of the coating application method and the storage temperature. Unfortunately, the negative influence played by the solvent solution masked this effect and prevented to obtain a significant improvement of the antioxidant activity over the negative control. However, we are confident that a better formulation of the chitosan solution would allow to obtain such an objective.

#### Influence of chitin deacetylation method

For the most of parameters analysed, chitosan obtained by heterogeneous deacetylation was significantly more effective than homogeneous chitosan in keeping the measured parameters more stable when the tomatoes were stored at RT, regardless of the coating application method. From the FTIR spectra, a lower deacetylation of the homogeneous chitosan compared to the heterogeneous one appeared. A lower effectiveness of the homogeneous method (operated at low temperatures) in removing acetyl groups from the chitin chain has been confirmed by other authors^60,61^. According to the literature, this may have negatively affected the barrier properties of the chitosan coating^62^. Under storage conditions at 4 °C, the effect of the two chitosan samples was similar in most cases. Probably the low temperature overcame the lower effectiveness of the homogeneous chitosan. However, the lower deacetylation of the homogeneous chitosan allowed a better efficiency of this coating in preserving the flavonoid concentration, probably because its structure was more similar to the original chitin. Chitosan is by itself a polymer more heterogeneous and complex than chitin, and the heterogeneous deacetylation gives a more complex mix of polymers than the homogeneous one. Since molecular size, degree and pattern of acetylation affects the elicitor activity^63^, the structural differences between the two chitosan polymers tested in this research may have influenced the recognition of these polysaccharides by the specific cell receptors.

#### Influence of coating application method

Results of this investigation generally showed that chitosan coatings applied by spraying were more effective in limiting the weight loss and the pH variation of tomatoes than those applied by dipping. An effect of the conditions of application on the coating properties, due to the different thickness of the coating, has been reported^63^. In the work by Leceta et al.^64^, firmness and texture in sprayed carrots were better than in the dipped ones. Dipped carrots also lost slightly more weight than the sprayed samples, similarly to the present results. On the contrary, the antimicrobial effect of the chitosan coating was greater when applied by dipping, due to the higher coating thickness obtained^63^. Hence, the method of applying the chitosan coating is another factor to be considered in this application. Other methods of application could also be investigated. For instance, brushing has been identified as a viable alternative for coating food products^39^.

## Conclusion

To the best of our knowledge, this is the first work that has investigated chitosan produced from an insect (*H. illucens*) as a coating for the preservation of a fresh food product. So far, only chitosan traditionally produced from crustacean exoskeletons had been used for this application. The results obtained showed a similar or even superior effect of chitosan deriving from *H. illucens* compared to the commercial one. Even if the chitosan coating often had no different effect than the untreated fruits, it gave better results than the coating with the solvent solution alone, leading to the hypothesis that a better formulation of the solvent solution is needed to allow the chitosan to function properly. Furthermore, insect-based chitosan was effective in reducing the alkalinisation of tomatoes at both RT and cold storage and, differently from the commercial polymer, it was generally able to contrast the negative effect played by the solvent solution on the phenolics and flavonoids content and on the antioxidant activity. It should be noted that in most of the literature studies, the effect of the coating with chitosan-free solution is not investigated, so that it is often uncertain whether the observed effects are due to the chitosan itself or whether the other reagents in the solution have an influence. In this work, the results obtained with the chitosan coating were always compared with a control consisting of the solvent-only coating solution, so that the significant effects observed are attributable to the chitosan itself. Further in-depth investigations are necessary to optimise both the production of chitosan from this insect biomass and its formulation as a coating solution for extending the shelf life of fresh products. For instance, different solvent solution compositions as well as further chitosan concentrations will need to be tested. The preliminary results presented in this work are an encouraging starting point and open to new opportunities for using a polymer of great economic interest derived from a waste product of the insect breeding chain.

## Materials and methods

### Materials

Mature, commercially available cherry tomatoes (*Solanum lycopersicum*) were purchased from a local grocery store in Potenza, Italy. Fruits of similar shape and colour, and without signs of fungal infection or mechanical damage were selected for experiments and after that they were immediately treated. Chitosan was produced from pupal exuviae of *H. illucens*, provided by Xflies s.r.l (Potenza, Italy). Commercial chitosan used as standard, and all the reagents used in the following experiments were purchased from Merck KGaA (Germany). All methods were carried out in accordance with institutional, national, and international guidelines and legislation.

### Chitin and chitosan preparation from *H. illucens* pupal exuviae

Pupal exuviae were dried in an oven at 60 °C for 48 h and ground into powder. Chitin was then extracted according to the method reported by Triunfo et al.^21^. Chitosan was produced from both unbleached and bleached chitin by two different approaches: heterogeneous deacetylation, referring to the work by Triunfo et al.^21^, and homogeneous deacetylation, according to the method by Hahn et al.^61^. Four different chitosan samples were obtained: heterogeneous unbleached and bleached chitosan, homogeneous unbleached and bleached chitosan.

### Characterization of chitin and chitosan

Chitin and chitosan from *H. illucens* were subjected to FTIR analysis using Jasco 460Plus IR spectrometer, in the range of wavelength 400–4000 cm^−1^. Functional groups identifying the polymers were defined from the resulting spectra.

Chitosan deacetylation degree (DD) was determined in accordance with Jiang et al*.*^65^, by potentiometric titration. Commercial chitosan with known DD was used as a reference to validate the method.

The viscosity-average molecular weight (M_v_) of chitosan samples was calculated by determining the intrinsic viscosity (η) using an Ostwald capillary type viscometer (Fisher Scientific, Waltham, Massachusetts, USA) following the method by Singh et al*.*^66^. The Mark–Houwink–Sakurada^67^ Eq. (1) was then used to calculate M_v_:

1 $$ [\upeta ] = KM_{\nu }^{ - \alpha } $$

where [η] is the intrinsic viscosity, K = 9.66 × 10^–5^ and α = 0.742.

The ability of chitosan derived from *H. illucens* to form a coating on the surface of tomatoes was investigated by assessing the film-forming capacity of the polymer. Briefly, a solution of 1% (w/v) chitosan in 1% (v/v) acetic acid was prepared per each chitosan sample and poured into a Petri dish. After drying at room temperature for 3 days, chitosan films were removed from the Petri dishes and photographically documented, visually evaluating their homogeneity and transparency.

### Chitosan-based coatings

#### Preparation and application of chitosan coating solutions

Chitosan-based coating solutions were prepared according to Hassan et al.^26^, dissolving the required amount of chitosan in 1% (v/v) acetic acid, with the addition of 0.2% (v/v) Tween-80 and 2% (v/v) glycerol to improve wettability. The solutions were left under continuous stirring for 16 h to allow dissolution of chitosan. The pH was then adjusted to 3.2 for all coating solutions. This pH level ensured that the chitosan remained in solution, avoiding its precipitation that would occur at more alkaline pHs. All the chitosan samples were tested at two different concentrations: 0.5% and 1% (w/v). A coating treatment with the solvent solution only (1% v/v acetic acid, 0.2% v/v Tween 80 and 2% v/v glycerol), and negative control of tomatoes without treatment were also considered. In addition to chitosan produced from pupal exuviae, a commercial chitosan sample was tested. The kinematic viscosity (ν) of the coating solutions was determined according to the standard method EN ISO 3104:2020^68^ by measuring the time for 7 ml of the solution to flow under gravity through a calibrated Ostwald glass capillary viscometer (Fisher Scientific, Waltham, Massachusetts, USA). Measurements were performed at 20 °C using water as reference liquid with known viscosity (1.002 mPa*s). The kinematic viscosity of the coating solutions was then calculated according to Eq. (2):

2 $$ \frac{{\nu_{l} }}{{\nu_{o} }} = \frac{{t_{l} \cdot \rho_{l} }}{{t_{o} \cdot \rho_{o} }} $$

where ν is the kinetic viscosity, t is the flow time, ρ is density, l is the solution to be measured and o is water used as reference.

Coating solutions were applied to tomatoes by two methods: dipping and spraying. For the dipping application, tomatoes were submerged in the coating solution for 5 min. Then, fruits were taken out and air-dried at RT for 30 min. This treatment was repeated three times. For the spraying application, the coating solutions were sprayed onto the fruits using an aerograph (Martellato s.r.l., Rovigo, Italy). Spraying of the fruit was repeated a second time after drying for 30 min at RT to ensure uniform surface coverage with the coating solution.

For each treatment, 36 tomatoes were used (18 coated by dipping and 18 coated by spraying), for a total of 432 fruits. Half of the fruits of each treatment were then stored at RT, while the other half were kept at 4 °C, for a total of 216 tomatoes per storage temperature (Supplementary table 8). The duration of the storage period was 30 days.

#### Determination of weight loss

All tomatoes were weighed regularly at an interval of 3 days for the whole duration of the storage period, using an electronic weighing balance. Weight loss was calculated comparing the initial and final weight of tomatoes, related to their original weight, and the results were expressed as percentage, according to the Eq. (3):

3 $$ Weight\;loss\;(\% ) = \frac{initial \, weight \, (g) \, - \, final \, weight \, (g)}{{initial \, weight \, (g)}} \times 100 $$

#### Total soluble solids (TSS) and pH determination

Pulp of tomatoes pooled in triplicates was homogenized and filtered through a gauze to remove solid residues and skins. 5 g of the filtered pulp were then suspended in 25 ml of distilled water and used for measurements. TSS were determined using a hand refractometer, according to the standard method EN ISO 2173:2003, and expressed as Brix°. The pH of the fruit pulp at room temperature was measured with a pH meter (Orion Research Inc., Boston, USA). TSS and pH measurements were performed at both the beginning and the end of the storage period, and results were expressed as percentage variation of these parameters.

#### Quantification of total phenolics and total flavonoids concentration

To perform further analyses, between the two chitosan concentrations, the 1% chitosan solution was selected since the two concentrations did not strongly impact the analysed physio-chemical parameters, and this concentration was the closest to the one used in Pagno et al.^7^ (1.5% of commercial chitosan). Fruit samples were extracted with 80% methanol by sonicating the mixture for 30 min, followed by 30 min stirring at 4 °C using a thermoshaker (Biosan, Riga, Latvia). The mixture was centrifuged (15 min, 14,000*g*) at 4 °C and the extraction procedure was repeated two additional times without the sonication step. The collected supernatants were pooled together and used for the determination of the total phenolic and flavonoid concentration and the antioxidant capacity.

Total phenolic concentration was determined with the spectrophotometric Folin–Ciocalteau method^69^, recording the absorbance at 750 nm and using a calibration curve of gallic acid (0–250 mg L^−1^).

Total flavonoids were quantified by the aluminium chloride colorimetric method as described by Kim et al*.*^70^, by recording the absorbance of the reaction mixture at 510 nm and using a standard curve of catechin (0–250 mg L^−1^).

#### Determination of antioxidant activity

Antioxidant activity was determined according to the methods described by Re et al*.*^71^, based on the ability of antioxidants present in the extract to reduce the preformed radicals of ABTS (2,2-azinobis (3-ethylbenzothiazoline-6-sulphonic acid)). Briefly, adequately diluted samples were added to ABTS solution to produce between 20 and 80% inhibition of the blank absorbance at 734 nm. A standard curve of Trolox (0–200 μmol L^−1^) was used to quantify the activity.

### Statistical analysis

All measurements were performed in triplicate and data expressed as average ± standard deviation. After assessing their non-normal distribution using the Shapiro–Wilk test, data were analysed by the Mann–Whitney *U* test. Statistical analyses were performed using JMP, Version 7 (SAS Institute Inc., Cary, NC, 1989–2021). Differences at *p* < 0.05 were considered significant.

## Supplementary Information

Supplementary Information.

## Data Availability

The datasets used and/or analysed during the current study are available from the corresponding author on reasonable request.
