# Supplementary material for: Preliminary investigation on the effect of insect-based chitosan on preservation of coated fresh cherry tomatoes
Source: Sci Rep. 2023 Apr 29;13:7030. doi: 10.1038/s41598-023-33587-0 (PMC10148861; doi:10.1038/s41598-023-33587-0)
Supplement: Supplementary file 1 — Supplementary Information. [file 41598_2023_33587_MOESM1_ESM.docx]

**Preliminary investigation on the effect of insect-based chitosan on preservation of coated fresh cherry tomatoes**

Elena Tafi^1^, Micaela Triunfo^1^, Anna Guarnieri^1^, Dolores Ianniciello^1^, Rosanna Salvia^1,2,^*, Carmen Scieuzo^1,2^, Annamaria Ranieri^3,4^, Antonella Castagna^3,4*^, Samuel Lepuri^3^, Thomas Hahn^5^, Susanne Zibek^5^, Angela De Bonis^1^, Patrizia Falabella^1,2^*

^1^ Department of Sciences, University of Basilicata, Potenza (Italy)

^2^ Spinoff XFLIES s.r.l, University of Basilicata, Potenza (Italy)

^3^ Department of Agriculture, Food and Environment, University of Pisa, Pisa (Italy)

^4^ Interdepartmental Research Center Nutrafood “Nutraceuticals and Food for Health”, University of Pisa (Italy)

^5^Fraunhofer Institute for Interfacial Engineering and Biotechnology IGB, Stuttgart (Germany)

*Corresponding authors: antonella.castagna@unipi.it; [r.salvia@unibas.it](mailto:r.salvia@unibas.it); [patrizia.falabella@unibas.it](mailto:patrizia.falabella@unibas.it)


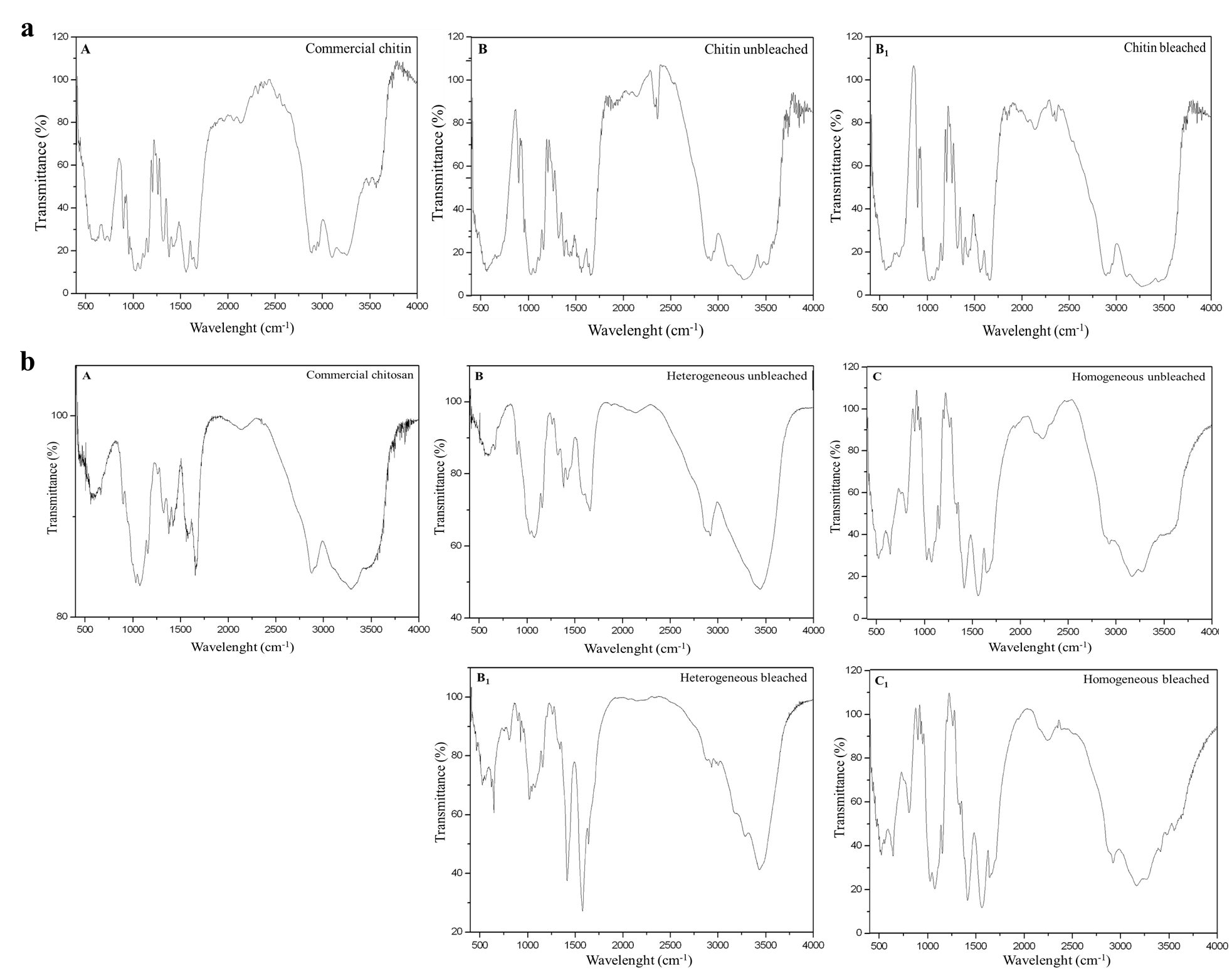


**Supplementary figure 1. (a)** **Spectra resulting from FTIR analysis of unbleached (B) and bleached (B_1_) chitin extracted from *H. illucens* pupal exuviae and commercial chitin produced from crustaceans (A). (b) Spectra resulting from FTIR analysis of unbleached (B, C) and bleached (B_1_, C_1_) chitosan samples produced from *H. illucens* pupal exuviae (heterogeneous (B, B_1_) and homogeneous (C, C_1_)) and commercial chitosan (A) derived from crustaceans.**


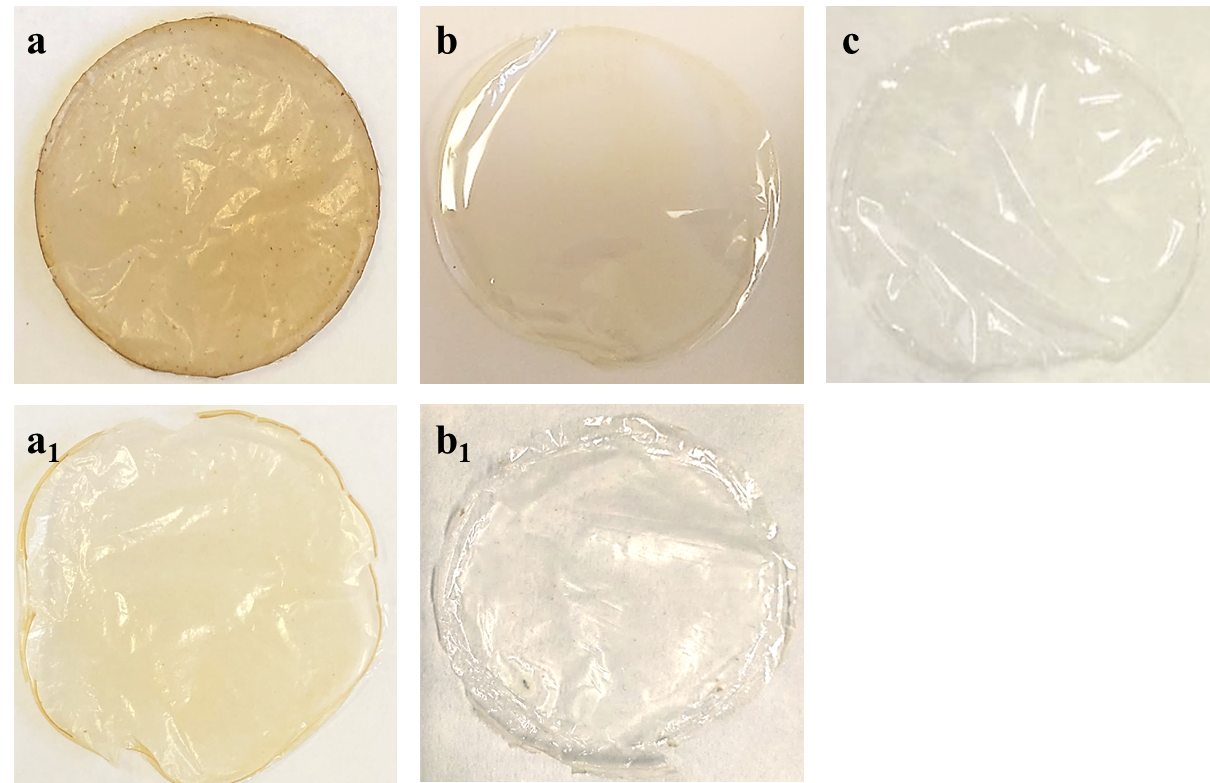


**Supplementary figure 2. Films obtained from the different chitosan samples produced from H. illucens pupal exuviae: heterogeneous unbleached (a), heterogeneous bleached (a_1_), homogeneous unbleached (b), homogeneous bleached (b_1_), and commercial chitosan derived from crustacean shells (c).**

**Supplementary table 1. Kinematic viscosity of chitosan-based and solvent-only coating solutions.**

| **Coating solution** | **Kinematic viscosity (mPa*s)** |
| --- | --- |
| Solvent | 1.08 |
| Comm CS 0.5% | 4.78 |
| Comm CS 1% | 8.67 |
| Het Unbl CS 0.5% | 4.00 |
| Het Unbl CS 1% | 6.67 |
| Het Bl CS 0.5% | 2.64 |
| Het Bl CS 1% | 3.86 |
| Hom Unbl CS 0.5% | 3.10 |
| Hom Unbl CS 1% | 5.31 |
| Hom Bl CS 0.5% | 2.30 |
| Hom Bl CS 1% | 3.62 |

**Supplementary table 2:** Weight loss (%) of tomatoes coated by dipping or spraying and stored at room or cold temperature for 30 days, expressed as mean ± standard deviation. P values resulting from the comparison of weight loss with the two different application methods are reported for each treatment, given the same storage temperature. Asterisks indicate significant differences (p<0.05), according to Mann-Whitney U test.

| TREATMENT | STORAGE TEMPERATURE | WEIGHT LOSS (%) | | *P* VALUE |
| --- | --- | --- | --- | --- |
|  |  | Dipping application | Spraying application |  |
| Solvent | RT | 38.2±2.7 | 59.3±4.3 | 0.05 |
|  | 4°C | 24.8±3.6 | 52.6±4.6 | 0.0007* |
| Comm CS 1 | RT | 35.3±3.3 | 28.9±2.4 | 0.14 |
|  | 4°C | 26.4±2.3 | 30.1±4.4 | 0.45 |
| Comm CS 0.5 | RT | 35.2±2.6 | 28.2±4.6 | 0.10 |
|  | 4°C | 24.5±1.3 | 22±4.9 | 0.20 |
| Het Unbl CS 1 | RT | 28.4±4.3 | 26.2±3.1 | 0.35 |
|  | 4°C | 20.4±3.1 | 24.9±2.8 | 0.03* |
| Het Unbl CS 0.5 | RT | 31.9±2.6 | 25.6±4.1 | 0.01* |
|  | 4°C | 24.6±2.5 | 28.1±3.7 | 0.50 |
| Het Bl CS 1 | RT | 25.7±3.9 | 23.8±3.6 | 0.35 |
|  | 4°C | 29.3±1.5 | 19.6±3.3 | 0.0003* |
| Het Bl CS 0.5 | RT | 28.6±2.9 | 26±4.2 | 0.20 |
|  | 4°C | 20.3±3.1 | 22.2±2.4 | 0.45 |
| Hom Unbl CS 1 | RT | 36.9±1.9 | 33.1±1.9 | 0.07 |
|  | 4°C | 21.6±3.1 | 23.8±3.1 | 0.23 |
| Hom Unbl CS 0.5 | RT | 36.5±4.8 | 30.6±2.8 | 0.01* |
|  | 4°C | 30±2.6 | 23.9±2.5 | 0.009* |
| Hom Bl CS 1 | RT | 42.3±4.4 | 40.1±2.3 | 0.56 |
|  | 4°C | 36.2±3.4 | 21.4±3.2 | 0.002* |
| Hom Bl CS 0.5 | RT | 35.3±3.3 | 30.9±3.3 | 0.04* |
|  | 4°C | 30.5±2.4 | 23.5±2.2 | 0.20 |

**Supplementary table 3:** Percentage increase of TSS of tomatoes coated by dipping or spraying and stored at room or cold temperature for 30 days, expressed as mean ± standard deviation. P values resulting from the comparison of weight loss with the two different application methods are reported for each treatment, given the same storage temperature. Asterisks indicate significant differences (p<0.05), according to Mann-Whitney U test.

| TREATMENT | STORAGE TEMPERATURE | TSS VARIATION (%) | | P VALUE |
| --- | --- | --- | --- | --- |
|  |  | Dipping application | Spraying application |  |
| Solvent | RT | 51.8±2 | 53.2±2.4 | 0.87 |
|  | 4°C | 21.1±0.8 | 73.9±3.3 | 0.003* |
| Comm CS 1% | RT | 44.6±1.7 | 35±1.7 | 0.26 |
|  | 4°C | 28.3±1.1 | 42.5±2 | 0.20 |
| Comm CS 0.5% | RT | 44.6±1.7 | 31.2±1.5 | 0.21 |
|  | 4°C | 28.3±1.1 | 38±1.8 | 0.36 |
| Het Unbl CS 1 | RT | 23.8±1.5 | 48.5±2.3 | 0.0008* |
|  | 4°C | 15.1±0.7 | 36.5±1.7 | 0.0012* |
| Het Unbl CS 0.5 | RT | 15±0.7 | 26±1.2 | 0.0064* |
|  | 4°C | 17.9±0.9 | 45.5±2.1 | 0.0015* |
| Het Bl CS 1 | RT | 9.2±0.4 | 32±1.5 | 0.021* |
|  | 4°C | 16.5±0.8 | 35±1.6 | 0.0019* |
| Het Bl CS 0.5 | RT | 31.1±1.5 | 32±1.5 | 0.48 |
|  | 4°C | 15±0.7 | 47±2.2 | 0.001* |
| Hom Unbl CS 1 | RT | 30.8±1.4 | 62±2.9 | 0.0011* |
|  | 4°C | 26.1±1.1 | 48.5±2.3 | 0.0007* |
| Hom Unbl CS 0.5 | RT | 53.2±2.4 | 59±2.8 | 0.10 |
|  | 4°C | 30.8±1.4 | 47±2.2 | 0.007* |
| Hom Bl CS 1 | RT | 62.8±2.8 | 74±3.5 | 0.015* |
|  | 4°C | 38.8±1.7 | 38±1.8 | 0.90 |
| Hom Bl CS 0.5 | RT | 35.6±1.6 | 65±3.1 | 0.0002* |
|  | 4°C | 26.1±1.5 | 45.5±2.1 | 0.0004* |

**Supplementary table 4:** Percentage variation of pH of tomatoes coated by dipping or spraying and stored at room or cold temperature for 30 days, expressed as mean ± standard deviation. P values resulting from the comparison of pH variation with the two different application methods are reported for each treatment, given the same storage temperature. Asterisks indicate significant differences (p<0.05), according to Mann-Whitney U test.

| TREATMENT | STORAGE TEMPERATURE | pH VARIATION (%) | | P VALUE |
| --- | --- | --- | --- | --- |
|  |  | Dipping application | Spraying application |  |
| Solvent | RT | 6.9±0.2 | 10.4±0.3 | 0.049* |
|  | 4°C | 4.2±0.1 | 15.7±0.5 | 0.049* |
| Comm CS 1% | RT | 7.2±0.2 | 5.1±0.1 | 0.51 |
|  | 4°C | 5.8±0.2 | 4.1±0.1 | 0.12 |
| Comm CS 0.5% | RT | 7.5±0.2 | 5±0.1 | 0.049* |
|  | 4°C | 11.1±0.3 | 2±0.1 | 0.049* |
| Het Unbl CS 1 | RT | 4.4±0.1 | 0 | 0.037* |
|  | 4°C | 2.1±0.1 | -4.2±0.1 | 0.049* |
| Het Unbl CS 0.5 | RT | 2.5±0.1 | 0 | 0.037* |
|  | 4°C | 1.7±0.1 | -3.1±0.1 | 0.049* |
| Het Bl CS 1 | RT | 11.1±0.3 | 2.9±0.1 | 0.049* |
|  | 4°C | 5.5±0.2 | 1.5±0 | 0.049* |
| Het Bl CS 0.5 | RT | 5.3±0.2 | 6.1±0.2 | 0.82 |
|  | 4°C | 0.9±0 | 0.3±0 | 0.046* |
| Hom Unbl CS 1 | RT | 8.5±0.2 | 6.4±0.2 | 0.13 |
|  | 4°C | 2.1±0.1 | 0.5±0 | 0.049* |
| Hom Unbl CS 0.5 | RT | 8.4±0.2 | 5.2±0.1 | 0.12 |
|  | 4°C | 3.5±0.1 | 0 | 0.037* |
| Hom Bl CS 1 | RT | 6.9±0.2 | 1.2±0 | 0.049* |
|  | 4°C | 0 | 1.2±0 | 0.12 |
| Hom Bl CS 0.5 | RT | 4.9±0.1 | 4.3±0.1 | 0.51 |
|  | 4°C | 0 | 1.2±0 | 0.12 |

**Supplementary table 5:** Total phenolics (TP) of tomatoes coated by dipping or spraying and stored at room or cold temperature for 30 days, expressed as mean ± standard deviation. *P* values resulting from the comparison of TP with the two different application methods are reported for each treatment, given the same storage temperature. Asterisks indicate significant differences (*p*<0.05), according to *Mann-Whitney* U test.

| TREATMENT | STORAGE TEMPERATURE | TP | | *P* VALUE |
| --- | --- | --- | --- | --- |
|  |  | Dipping application | Spraying application |  |
| Solvent | RT | 0.352 ± 0.024 | 0.442 ± 0.031 | 0.0495* |
|  | 4°C | 0.287 ± 0.0640 | 0.246 ± 0.025 | 0.275 |
| Comm CS 1 | RT | 0.299 ± 0.030 | 0.333 ± 0.072 | 0.513 |
|  | 4°C | 0.370 ± 0.060 | 0.295 ± 0.005 | 0.0495* |
| Het Unbl CS 1 | RT | 0.517 ± 0.031 | 0.539 ± 0.038 | 0.513 |
|  | 4°C | 0.361 ± 0.030 | 0.373 ± 0.054 | 0.513 |
| Het Bl CS 1 | RT | 0.435 ± 0.016 | 0.542 ± 0.034 | 0.0495* |
|  | 4°C | 0.309 ± 0.006 | 0.347 ± 0.030 | 0.513 |
| Hom Unbl CS 1 | RT | 0.608 ± 0.032 | 0.633 ± 0.003 | 0.513 |
|  | 4°C | 0.484 ± 0.008 | 0.485 ± 0.053 | 0.827 |
| Hom Bl CS 1 | RT | 0.696 ± 0.039 | 0.597 ± 0.039 | 0.0495* |
|  | 4°C | 0.559 ± 0.055 | 0.473 ± 0.045 | 0.0495 |

**Supplementary table 6:** Total flavonoids (TF) of tomatoes coated by dipping or spraying and stored at room or cold temperature for 30 days, expressed as mean ± standard deviation. *P* values resulting from the comparison of TF with the two different application methods are reported for each treatment, given the same storage temperature. Asterisks indicate significant differences (*p*<0.05), according to *Mann-Whitney* U test.

| TREATMENT | STORAGE TEMPERATURE | TF | | *P* VALUE |
| --- | --- | --- | --- | --- |
|  |  | Dipping application | Spraying application |  |
| Solvent | RT | 0.060 ± 0.005 | 0.074 ± 0.005 | 0.0495* |
|  | 4°C | 0.045 ± 0.009 | 0.039 ± 0.016 | 0.827 |
| Comm CS 1 | RT | 0.063 ± 0.001 | 0.078 ± 0.016 | 0.246 |
|  | 4°C | 0.060 ± 0.018 | 0.057 ± 0.005 | 0.827 |
| Het Unbl CS 1 | RT | 0.068 ± 0.008 | 0.068 ± 0.003 | 1 |
|  | 4°C | 0.070 ± 0.014 | 0.060 ± 0.011 | 0.275 |
| Het Bl CS 1 | RT | 0.101 ± 0.004 | 0.062 ± 0.002 | 0.0495* |
|  | 4°C | 0.041 ± 0.010 | 0.046 ± 0.015 | 0.827 |
| Hom Unbl CS 1 | RT | 0.140 ± 0.007 | 0.146 ± 0.001 | 0.246 |
|  | 4°C | 0.081 ± 0.002 | 0.077 ± 0.007 | 0.513 |
| Hom Bl CS 1 | RT | 0.133 ± 0.022 | 0.138 ± 0.009 | 0.513 |
|  | 4°C | 0.058 ± 0.003 | 0.059 ± 0.001 | 0.658 |

**Supplementary table 7:** Antioxidant activity (AA) of tomatoes coated by dipping or spraying and stored at room or cold temperature for 30 days, expressed as mean ± standard deviation. *P* values resulting from the comparison of AA with the two different application methods are reported for each treatment, given the same storage temperature. Asterisks indicate significant differences (*p*<0.05), according to *Mann-Whitney* U test.

| TREATMENT | STORAGE TEMPERATURE | AA | | *P* VALUE |
| --- | --- | --- | --- | --- |
|  |  | Dipping application | Spraying application |  |
| Solvent | RT | 0.598 ± 0.074 | 0.823 ± 0.061 | 0.0495* |
|  | 4°C | 0.862 ± 0.219 | 0.534 ± 0.112 | 0.127 |
| Comm CS 1 | RT | 0.578 ± 0.033 | 0.668 ± 0.068 | 0.127 |
|  | 4°C | 0.802 ± 0.175 | 0.651 ± 0.061 | 0.275 |
| Het Unbl CS 1 | RT | 0.667 ± 0.083 | 0.679 ± 0.038 | 0.827 |
|  | 4°C | 0.897 ± 0.148 | 0.708 ± 0.123 | 0.127 |
| Het Bl CS 1 | RT | 1.021 ± 0.099 | 0.594 ± 0.038 | 0.0495* |
|  | 4°C | 0.548 ± 0.102 | 0.590 ± 0.131 | 0.513 |
| Hom Unbl CS 1 | RT | 1.436 ± 0.129 | 1.773 ± 0.106 | 0.0495* |
|  | 4°C | 1.215 ± 0.096 | 0.858 ± 0.162 | 0.0495* |
| Hom Bl CS 1 | RT | 1.463 ± 0.005 | 1.384 ± 0.074 | 0.0495* |
|  | 4°C | 1.361 ± 0.106 | 1.120 ± 0.158 | 0.127 |

**Supplementary table 8. Number of tomatoes used per each treatment, coating application method (dipping or spraying) and storage temperature (RT or 4°C).** Treatments consisted of untreated fruits (Ctrl -), solvent only, and coating with the different chitosan (CS) samples: commercial (Comm), heterogeneous unbleached (Het Unbl), heterogeneous bleached (Het Bl), homogeneous unbleached (Hom Unbl) and homogeneous bleached (Hom Bl).

| Treatment | [CS] (%) | Dipping-coated | | Spraying-coated | | Total fruits |
| --- | --- | --- | --- | --- | --- | --- |
|  |  | RT | 4°C | RT | 4°C |  |
| Ctrl - | - | 9 | 9 | 9 | 9 | 36 |
| Solvent | - | 9 | 9 | 9 | 9 | 36 |
| Comm CS | 0.5% | 9 | 9 | 9 | 9 | 36 |
|  | 1% | 9 | 9 | 9 | 9 | 36 |
| Het Unbl CS | 0.5% | 9 | 9 | 9 | 9 | 36 |
|  | 1% | 9 | 9 | 9 | 9 | 36 |
| Het Bl CS | 0.5% | 9 | 9 | 9 | 9 | 36 |
|  | 1% | 9 | 9 | 9 | 9 | 36 |
| Hom Unbl CS | 0.5% | 9 | 9 | 9 | 9 | 36 |
|  | 1% | 9 | 9 | 9 | 9 | 36 |
| Hom Bl CS | 0.5% | 9 | 9 | 9 | 9 | 36 |
|  | 1% | 9 | 9 | 9 | 9 | 36 |
| Total fruits |  | 108 | 108 | 108 | 108 | 432 |
